# Loose Semirigid Aromatic Polyester Bottle Brushes at Poly(2-isopropyl-2-oxazoline) Side Chains of Various Lengths: Behavior in Solutions and Thermoresponsiveness

**DOI:** 10.3390/polym14245354

**Published:** 2022-12-07

**Authors:** Elena Tarabukina, Anna Krasova, Mikhail Kurlykin, Andrey Tenkovtsev, Alexander Filippov

**Affiliations:** Institute of Macromolecular Compounds, Russian Academy of Sciences, 199004 Saint-Petersburg, Russia

**Keywords:** graft copolymers, aromatic polyester, poly(2-isopropyl-2-oxazoline), thermoresponsiveness, phase separation, dilute solutions, molecular characteristics, light scattering, dynamic light scattering, turbidimetry

## Abstract

A polycondensation aromatic polyester with an oxygen spacer was synthesized and used as a macroinitiator for the grafting of linear poly(2-isopropyl-2-oxazoline) (PiPrOx) by the cationic polymerization method. The length of the thermosensitive side chains was varied by the initiator:monomer ratio. Using methods of molecular hydrodynamics, light scattering and turbidimetry, the copolymers were studied in organic solvents and in water. The molecular characteristics of the main chain and graft copolymers, the polymerization degree of side chains and their grafting density have been determined. The equilibrium rigidity of the macroinitiator and the conformations of grafted macromolecules were evaluated. In selective solvents, they take on a star-like conformation or aggregate depending on the degree of shielding of the main chain by side chains. The thermoresponsiveness of graft copolymers in aqueous solutions was studied, and their LCST were estimated. The results are compared with data for graft copolymers composed of PiPrOx side chains and flexible or rigid chain backbones of aromatic polyester type.

## 1. Introduction

Grafted polymers, the so-called cylindrical polymer brushes, are a class of polymers consisting of a long main chain and relatively short side chains grafted to it [1]. The physicochemical properties of molecular brushes in solution and in block are largely determined by their architectural parameters, such as the degree of polymerization of the main and side chains, the grafting density of side chains, etc. [1,2,3,4,5,6,7,8]. Due to the peculiarities of molecular architecture, cylindrical brushes are widely used to design new nanoobjects of various morphologies that cannot be obtained on the basis of linear polymers [9,10,11].

Research into molecular brushes began at the end of the 20th century due to the development of controlled synthesis of polymers of various architectures [2,3,12,13]. As a result, the main regularities of the behavior of graft copolymers in solutions, such as the effect of the length of the main and side chains and the grafting density of the latter, on the hydrodynamic characteristics and conformation of their macromolecules have been established [5,6,7,8,9,14,15,16,17,18,19,20].

The properties of grafted block-copolymers can depend on the interaction of chemically dissimilar side and main chains and on the solvent affinity to different blocks of the graft copolymer [4,13,21,22,23,24,25,26]. Accordingly, one of the ways to control the self-organization of molecular brushes in selective solvents is the targeted synthesis of brushes with different chemical structures of the main and side chains and molecular architecture parameters. The self-organization of amphiphilic polymer brushes differs significantly from the behavior of linear copolymers in solutions [27,28,29,30]. For example, amphiphilic polymer brushes in selective solvents make it possible to obtain monomolecular micellar core–shell structures, which can serve for the transport and isolation of inorganic particles in solution. For solutions of amphiphilic aromatic polyimides grafted onto vinyl polymers such as polystyrene, poly(methyl-methacrylate), poly(tert-butyl-acrylates) and their copolymers with poly(methacrylic acid), it has been shown that copolymers with a low grafting density form aggregates due to contacts with polyimide backbones. At high grafting density and longer side chains, brush conformations change from spherical in theta solvents to rod-like in good solvents [24,31,32,33].

One of the promising trends in the development of nanostructured polymeric materials is the synthesis of complex polymeric associated systems capable of reversibly changing the hydrophilic-hydrophobic balance under external impacts, such as temperature, pH, irradiation, etc. Under conditions, such “smart” systems can modify their intra- and intermolecular organization, as well as complex-forming properties. The stimuli-responsive properties can be controlled by varying the chemical structure, molecular architecture, and composition of the graft copolymer [34,35,36,37,38]. For example, for graft copolymers with poly(N-isopropylacrylamide) side chains, a significant effect of the end group structure and side chain length on the behavior of aqueous solutions upon heating was revealed [36]. An important role in the formation of the properties of molecular brushes in solutions is also played by the rigidity of their structural elements, main and side chains. The presence of rigid chain fragments in side chains can lead to their orientational ordering in solution [4]. On the other hand, grafting of long, flexible chains onto a rigid aromatic backbone prevents aggregation when the grafting is sufficiently dense [39].

To obtain reversibly thermosensitive systems, polymers based on poly(N-isopropylacrylamide), dimethyl-amino-ethyl-acrylates, oligo(ethylene-oxide), and poly(2-alkyloxazolines) (PAlOx) are used [40]. PAlOx are especially in demand for medical applications due to their biocompatibility and rather low LCST (i.e., the lower critical solution temperature) [41]. Tertiary amide groups in PAlOx ensure their stability in biological media [42,43,44,45]. Complexes of PAlOx and their copolymers with low molecular weight compounds and metal ions are promising for drug and DNA delivery [46], for the design of biocompatible composite structures [47] and other applications. PAlOx graft copolymers have been shown to be the most promising matrices for drug delivery [48].

To date, the effect of the PAlOx copolymer architecture, namely, their star-shaped, grafted or block structure, the chemical nature of the end groups and the hydrophobic-hydrophilic balance, on the temperature dependence of the polymer solubility has been studied [49,50,51,52,53,54,55]. For methacrylic acid copolymers or polystyrene derivatives as a backbone, it has been shown that the cloud point *T*_cp_ decreases with increasing molecular weight and backbone length [50]. Graft copolymers with methacrylate backbone and poly(2-ethyl-2-oxazoline) (PEtOx) side chains demonstrated the coexistence of aggregates and macromolecules at low temperatures [49].

Amphiphilic thermoresponsive PEtOx block copolymers with an aromatic polyester (APE) backbone have been studied to a lesser extent. Such copolymers can be obtained by a polymerization-polycondensation reaction, in which a main chain is formed as a result of polycondensation, followed by grafting from polymerization of side chains [56,57,58]. Most solvents for such macromolecules are selective. The authors of [59] proposed the synthesis of a polycondensation APE macroinitiator containing functional groups suitable for initiating the cationic polymerization of 2-alkyl-2-oxazolines to obtain graft copolymers. Using the polymerization-polycondensation approach, stimulus-sensitive graft copolymers (APE_6_) with an aromatic polyester backbone flexible main chain containing a –(CH_2_)_6_– as a spacer and PEtOx side chains (APE_6_-g-PEtOx) were synthesized [60,61]. For APE_6_-g-PEtOx, it was shown that the side chain grafting density *z* determines the mechanism of macromolecules self-organization, namely, compaction or aggregation, and causes a change in the phase separation temperature, which increases with *z*. The replacement of side chains with more hydrophobic poly(2-isopropyl-2oxazoline) (PiPrOx) leads to a decrease in phase separation temperatures [62]. Note that dehydration of 2-ethyl-2-oxazoline units begins at 50 °C, and that of 2-isopropyl-2-oxazoline at about 20 °C [63]. Cloud points observed for PiPrOx are generally higher than for the 2-isopropyl-2-oxazoline monomer; the thermal transition temperature of PiPrOx is affected by polymer concentration and molar mass, as well as terminal functional groups. When varying these parameters, *T*_cp_ have been observed by various studies at temperatures from 36 to 63 °C. Molecular architecture can also influence *T*_cp_: for example, a cyclic PiPrOx exhibited *T*_cp_ around 10 °C higher than a linear PiPrOx of the same molar mass. [40,41,64]. It has been shown that, in a selective solvent, APE_8_-g-PiPrOx polymerization-polycondensation molecular brush with short PiPrOx chains and a backbone containing sufficiently long alkylene fragments –(CH_2_)_8_– in the monomer unit forms unimolecular micelles of a star-like conformation at a low *z* ~0.5. On the contrary, molecular brushes with PAlOx side chains and the rigid main chain of aromatic APE (APE_r.ch._-g-PAlOx) at a low *z* and short side chains aggregate into large supramolecular structures, and they are molecularly dispersed only at sufficiently long side chains [65]. In this case, the LCST is affected by the chemical structure of the side chains.

**Figure 1:**
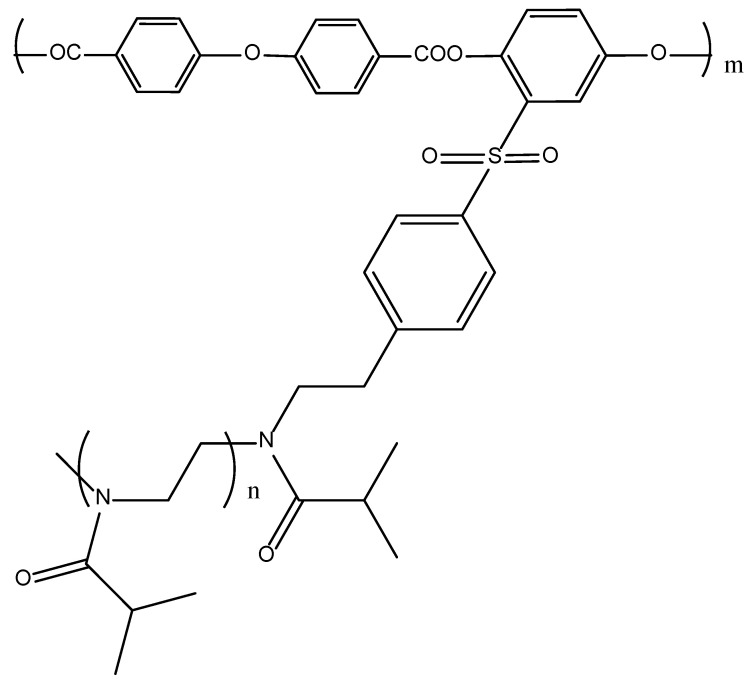
Chemical structure of APE_O_-g-PiPrOx.

Thus, the chemical structure and equilibrium rigidity of the main chain essentially determine the mechanism of self-organization of a molecular brush in solution. It seems important to continue the series of copolymers by varying the structure of the main chain, passing to a main chain with an intermediate equilibrium rigidity. For this purpose, an aromatic polyester type macroinitiator with an oxygen atom as a spacer was synthesized (APE_O_) and graft copolymers with PiPrOx side chains of various lengths were obtained (Figure 1). Thus, the main objectives of this work were to determine the hydrodynamic and conformational characteristics of the APE_O_ macroinitiator and its block copolymers with grafted PiPrOx chains, and to study the effect of the side chain length on the conformational characteristics of APE_O_-g-PiPrOx copolymers in selective solvents and on thermal responsiveness in aqueous solutions.

## 2. Experimental

### 2.1. Materials and Instruments

2-[4-(2-Br-ethyl)]phenylsulfonyl hydroquinone [66], 4,4′-oxydibenzoyldicloride [67], 2-isopropyl-2-oxazoline [68] were obtained according to the known procedures. Diphenyl oxide, 1-chloronaphalene and 1,1,2,2-tetrachloroethane (Aldrich) were dried over calcium hydride and distilled. NMR spectra of solutions of samples in CDCl_3_ were recorded using a Bruker AC 400 instrument (400 MHz). Dialysis was performed with the use of dialysis bags (CellaSep, Orange Scientific) with MWCO 3500 D. Chromatographic analysis was performed with the use of a Shimadzu LC-20AD chromatograph equipped with a TSKgel G5000HHR column (5 μm, 7.8 mm × 300 mm, Tosoh Bioscience) and a refractometric detector. Solution of LiBr in DMF (0.1 mol/L) at 60 °C was used as a mobile phase. Calibration was performed with the use of poly(ethylene glycol) standards (*M*_w_ = 6 × 10^2^–4 × 10^4^).

### 2.2. Synthesis of Poly(2-[4-(2-Br-ethyl)phenylsulfonyl]-1,4-phenylene-4′,4″-oxydibenzoate

A flask equipped with a stirrer and a gas-supplying tube was charged with 2-[4-(2-Br-ethyl)]phenylsulfonyl hydroquinone (4.23 g, 0.01 mol), 4,4′-oxydibenzoyl chloride (2.95 g, 0.01 mol), and diphenyl oxide (30 mL). The obtained mixture was purged with dry argon and heated up to 200 °C. The reaction mixture was kept at 200 °C for 2 h. The polymer was precipitated with hexane, filtered off, reprecipitated from chloroform into hexane, and dried to a constant mass. The yield was 5.9 g (92%). ^1^H NMR (CDCl_3_): 3.22 (d, ArCH_2_CH_2_Br), 3.56 (d, ArCH_2_CH_2_Br), 7.11–8.43 (m, Ar–H) ppm. [η] = 0.24 dL/g (CHCl_3_, 25 °C); M_w_ = 23,000.

### 2.3. Synthesis of Polyester-g-poly-2-isopropyl-2-oxazoline Copolymers

Solution of the initiator (30 wt.%) and the monomer in 1,1,2,2-tetrachloroethane (feed ratio of about 10% from the desired value) was heated in sealed tube at 100 °C for 3 h. Then, the rest of the monomer was added to reaction mixture and polymerization was allowed to proceed at 120 °C for 24 h. After completion of polymerization and removal of volatile compounds, the reaction mixture was diluted with ethanol, dialyzed against water for 48 hrs and lyophilized.

### 2.4. Determination of the Molecular Characteristics of Polymers

The weight-average molar masses *M*_w_ of the macroinitiator and copolymers and the hydrodynamic radii *R*_h_ of their macromolecules were determined in dilute solutions in organic solvents (chloroform, nitropropane) by static (LS) and dynamic (DLS) light scattering methods. Since there was practically no asymmetry in the scattered light intensity, *M*_w_ was determined by the Debye method. The equation [69]:

(1) $$\frac{cH}{I_{}}=\frac{1}{M_{w}}+2A_{2}c$$

where *I* is the intensity of light scattering measured at 90°, *A_2_* is the second virial coefficient, and *H* is the optical constant,

(2) $$H=\frac{4\pi^{2}n_{{}_{0}}^{2}{\left(dn/dc\right)}^{2}}{N_{A}\lambda_{0}^{4}},$$

*N_A_* being the Avogadro’s number was applied. The refractive index increment dn/dc was measured on a Refractometer RA-620/600 (KEM, Kyoto, Japan), with LED Na-D Line light source. The LS and DLS measurements were carried out in a Photocor Complex set-up (Photocor Instrument, Moscow, Russia), which was equipped with a Photocor-PC2 correlator with 288 channels, and a Photocor-PD detector for measuring the transmitted light intensity. The light source was a Photocor-DL semiconductor laser with a wavelength of λ_0_ = 659.1 nm. Calibration was carried out with toluene, the absolute scattering intensity being *R*_v_ = 1.38 × 10^−5^ cm^−1^. Before measurements, the solutions were filtered into dust-free cells using Chromafil polyamide filters (Macherey-Nagel GmbH & Co. KG) with a pore size of 0.45 μm.

The hydrodynamic radius *R*_h_ was determined by the regularization method which is used as a part of the Photocor Complex software. The measurements were taken at an angle of 90 degrees.

Intrinsic viscosity [η] was measured in Ostwald type Cannon-Manning capillary viscometers (Cannon Instrument Company, State College, PA, USA) and an LOIP LT-100 temperature control unit (LOIP, St.-Petersburg, Russia). Viscosity data were analyzed using the Huggins equation

η_sp_/*c* = [η] + *k*′[η]^2^*c*, (3)

where *k*′ is the Huggins constant characterizing the polymer–solvent hydrodynamic and thermodynamic interactions in solutions and η_sp_ = (τ/τ_0_ − 1), τ being the solution flow time [70,71]. The solvent efflux time was *τ*_0_ = 116.0 s for chloroform, 105.4 s for nitropropane and 57.2 s for DMF.

### 2.5. Study of Thermoresponsiveness of Copolymers in Solutions upon Heating

The thermoresponsiveness of copolymers was studied by LS, DLS and turbidimetry in aqueous solutions using the Photocor instrument described above. The temperature *T* was controlled with an accuracy of 0.1 °C, changing it discretely with a step of 1.0 °C near the phase separation point *T*_1_ and with a larger interval up to 5.0–6.0 °C at temperatures far from *T*_1_.

Scattered light intensity *I* and transmitted light intensity I* were measured as functions of *T* upon heating. The temperatures *T_1_* and T*_1_ of the onset of phase separation were determined from the dependences *I(T)* and I*(T), taking for *T_1_* and T*_1_ the temperatures after which *I* began to increase or I* decreased, respectively. The hydrodynamic radii of scattering species *R_i_* and their contribution *S_i_* to the total scattering intensity *S*, *i* being associated with the type of scattering specie, were also measured at various *T*. The *S_i_* value was estimated as the area under the corresponding peak of *I(R)* distribution obtained by DLS. It should be noted that measurements of all parameters were carried out after the solution reached an equilibrium state, that is, when the *I* and I* reached constant values after an abrupt change in *T*.

## 3. Results and Discussion

### 3.1. Synthesis of Multicenter Oligoester Initiator

In the synthesis of the macroinitiator, high-temperature solution polycondensation without acceptor was used [72]; this method allows synthesize polyesters over a wide range of molecular masses with polydispersity index of 2.1–2.3. Synthesis of the APE_O_ macroinitiator was conducted in accordance with that described in [59]. The conditions for the polycondensation were the following: 1-chloronaphthalene as the solvent; temperature of 200 °C, monomer concentration 25 wt.%, reaction time 2 h.

### 3.2. Synthesis of APE_O_-g-PiPrOx with the Use of APE_O_ Macrointiator

**Figure 2:**
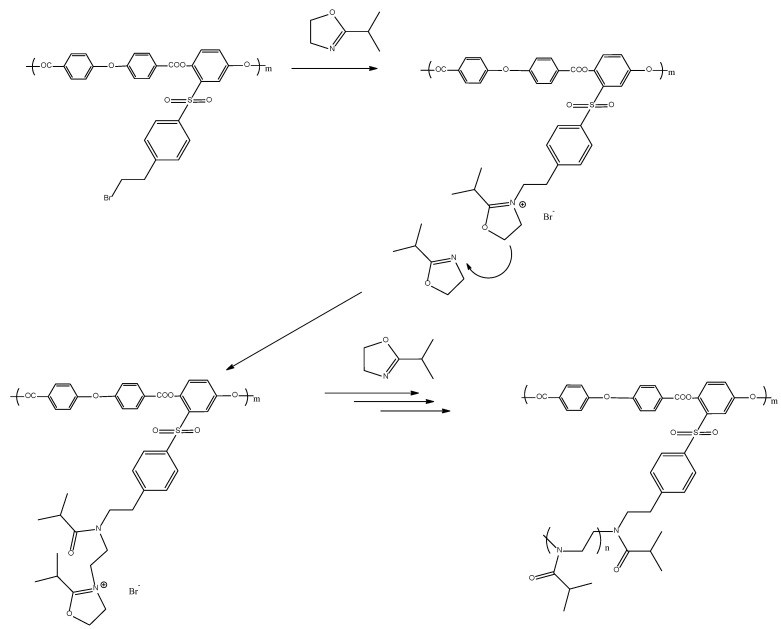
Synthesis of APE_O_-g-PiPrOx graft copolymers.

**Figure 3:**
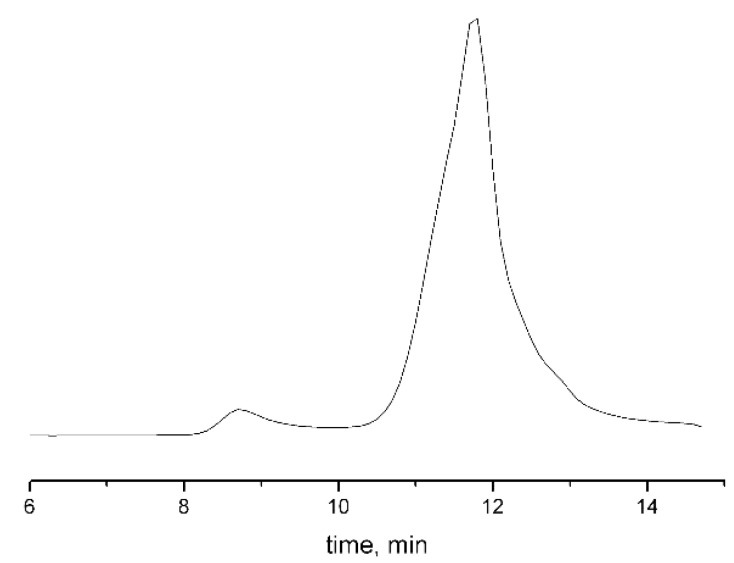
Eluogram for APE_O_-g-PiPrOx sample obtained after grafting at 1/40 initiator/monomer ratio.

Methods and approaches to the preparation of APE_O_-g-PiPrOx block copolymers are described in [59]. Graft copolymers were synthesized via CROP of 2-isopropyl-2-oxazoline according to the following scheme (Figure 2):

Grafting of PiPrOx chains was carried out in tetrachloroethane solution at 120 °C to improve solubility of the macroinitiator monomer, since thermodynamic quality of a solvent becomes higher with increasing temperature. The process was carried out in two stages. In the first stage, 10% from the calculated amount of the monomer was introduced into polymerization mixture, and the mixture was heated at 100 °C for 3 h. The addition of the remaining amount of oxazoline made it possible to post-polymerize the side chains due to the “living” character of the process. When loading the reaction mixture, the initiator/monomer ratio was 1/40 (sample 1), 1/100 (sample 2) and 1/120 (sample 3), which made it possible to obtain three samples of the graft copolymer with PiPrOx side chains of various lengths. GPC data confirm unimodality in their size distribution (Figure 3). The amount of grafted PiPrOx was estimated using ^1^H NMR data by the ratio of signal intensity of the terminal methyl group of 4-methylpiperidine (at about 0.9 ppm) and signal intensity of poly(ethylene imine) fragments (at about 3.4 ppm). The average degree of polymerization of monomer units *N*_s_ in grafted oligo(oxazoline) chains was 30, when the initial initiator/monomer ratio was equal to 1/40; this value was 50 at a ratio of 1/100 and 60 at a ratio of 1/120. The fact that degree of polymerization of grafted chains is not proportional to initial initiator/monomer ratio is caused by chain transfer to monomer. This issue was analyzed by us earlier [59]. The observed decrease in initiation efficiency in the case of multicenter macroinitiators may be due to higher growth rate of side chains than the initiation rate; therefore, steric hindrances of side chains impede the process. Indeed, the asymmetric shape of the chromatogram obtained for graft copolymers (Figure 3) indicates some inhomogeneity in distribution of grafted fragments alongside the backbone.

### 3.3. Molecular and Conformational Properties of APE_O_ and APE_O_-g-PiPrOx

**Figure 4:**
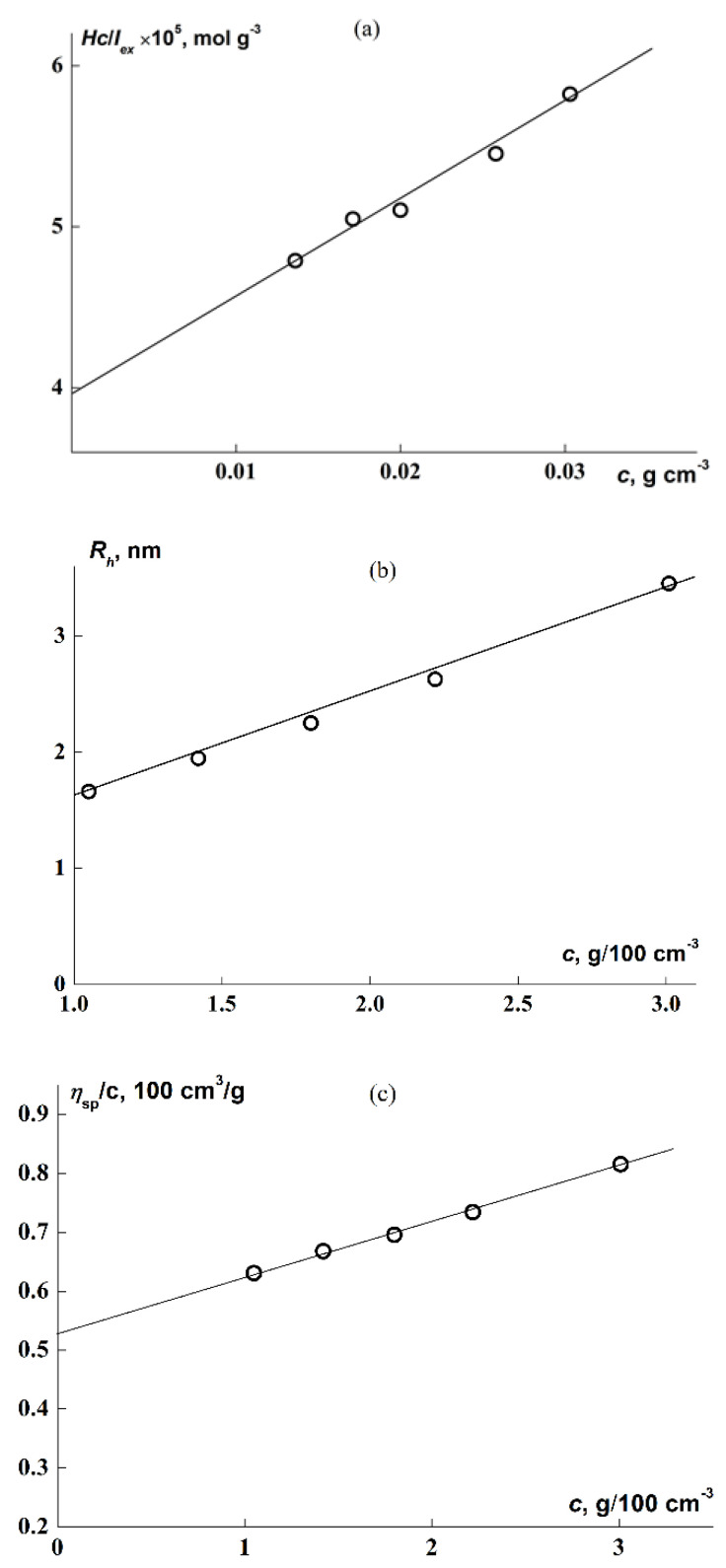
Concentration dependences of *cH/I_ex_* (**a**), *R*_h_ (**b**), and η_sp_/*c (***c***)* for APE_O_ solutions in CHCl_3_.

The molecular and hydrodynamic characteristics of the macroinitiator were determined in chloroform. Graphs for their determination are presented in Figure 4; Table 1 presents the results. The values of the second virial coefficient *A*_2_ and the Huggins constant *k*′ calculated from plots of Figure 4a,c (Equations (1)–(3)) testify to the good thermodynamic quality of CHCl_3_ as a solvent for APE_O_. The *R*_h_ was obtained by extrapolating the hydrodynamic radii determined at finite concentrations to *c* = 0 (Figure 4b). The viscometric hydrodynamic radius *R*_η_ = 5.7 nm for APE_O_, calculated from the formula

*R*_η_ = (3*M*[η]/10πN_A_)^1/3^, (4)

significantly exceeds *R*_h_, which may testify to an extended (asymmetric) conformation of the macroinitiator. The polymerization degree of the macroinitiator calculated from the *M*_w_ value (Table 1) is *N*_0_ = 38, since the molar mass of the APE_O_ monomer unit is *M*_0_ = 580 g/mol. To estimate the length of the monomer unit of the macroinitiator *L*_0_, it was assumed that the length of valence bonds is 0.14 nm and the bond angles are tetrahedral; then, in accordance with the APE_O_ structural formula, the sum of its bond lengths along the chain direction is *L*_0_ = 1.6 nm (without deformation of the bond angles) and the macromolecular chain length is *L* = 60.7 nm.

The equilibrium rigidity, which is measured by the Kuhn segment length *A*, for an APE_O_ can be estimated by comparing its characteristics with the *A* value for a polymer of close chemical structure, for example, semi-rigid poly(m-phenylene isophthalamide) (PMPhIPhA), studied in [73]. For this polymer, the values *A* ≈ 4–4.7 nm were obtained, which are increased, compared to flexible-chain polymers, due to aromatic fragments in the meta position. Although the comparison of structures of APE_O_ and PMPhIPhA is quite approximate, it can be assumed that the equilibrium rigidity of APE_O_ should not be greater than that of PMPhIPhA, since the –O– spacer present in the structure of APE_O_ allows rotation of the chain around the valence bond; thus, Kuhn segment length for APE_O_ should be 2 nm < *A* < 4 nm, where *A* = 2 nm was obtained for APE macroinitiators with flexible alkylene spacers [74]. Then the APE_O_ chain consists of more than 15 Kuhn segments, therefore it should be sufficiently coiled in solution. Thus, a worm-shaped cylinder with a contour length of 60.7 nm can serve as a model for the APE_O_ macroinitiator chain in solution. The increased equilibrium rigidity of APE_O_ and, consequently, the increased permeability of its macromolecules in NP probably cause their low *R*_h_ (Table 1).

**Figure 5:**
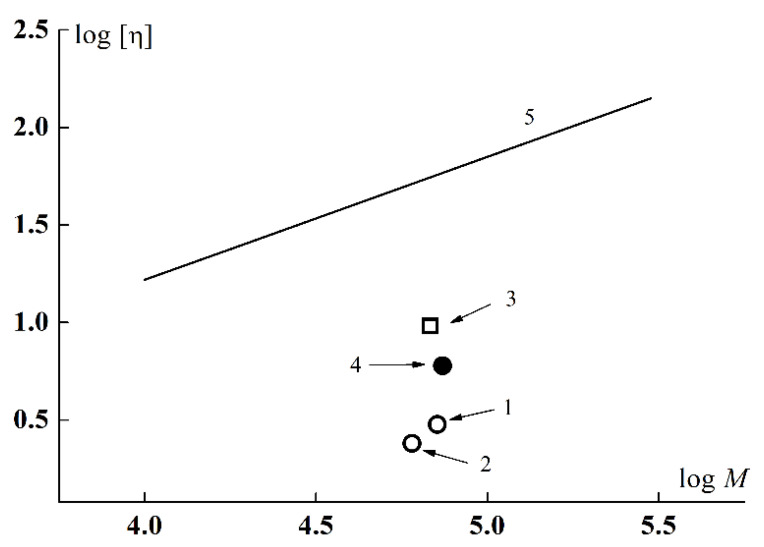
Intrinsic viscosity [η] vs. *M*_w_ for APE_O_-g-PiPrOx sample 1 (1) and sample 2 (2) in nitropropane, APE_O_-g-PiPrOx sample 3 in DMF (3), APE_8_-g-PiPrOx [62] (4), and Mark–Kuhn–Houwink–Sakurada dependence [η] = 5.0 × 10^−2^M^0.63^ given for linear PEtOx [75] (5).

*M*_w_ and hydrodynamic characteristics of APE_O_-g-PiPrOx graft copolymers were determined in nitropropane (NP) (Table 1). Debye plots for determining *M*_w_ are shown in Figure S1. The *A*_2_ values are positive and decrease with increasing iPrOx monomer content indicating that the solvent is worsening for the copolymers upon the initiator/monomer ratio. The hydrodynamic radii obtained by the DLS method did not depend on the concentration, and Table 1 presents the concentration-averaged *R*_h_ values. The hydrodynamic radii of APE_O_-g-PiPrOx exceed the *R*_h_ obtained for the macroinitiator (Table 1). This increase is conditioned by significant contribution of side chains to the friction coefficient of the macromolecule in the solvent. However, the values of both *R*_h_ and intrinsic viscosities [η] are very small at sufficiently large *M*_w_, which can be seen from the Figure 5. Here, the points corresponding to the data for samples 1 and 2 in NP lie well below the logarithmic dependence [η](*M*) carried out for linear PEtOx according to the equation [η] = 5.0 × 10^−2^ M^0.63^ from the work [75], and also lower than for APE_8_-g-PiPrOx in NP [62]. This testifies to a high intramolecular density of APE_O_-g-PiPrOx macromolecules. As was shown in [32,62], the grafted block-copolymer can adopt a star-like conformation, in case of low grafting degree of side chains and short grafted blocks; here the arms are the PiPrOx side chains and the core is the backbone macromolecule collapsed in a nonsolvent. The [η] and *R*_h_ values of sample 3 were measured in DMF (Table 1). They noticeably exceed [η] and *R*_h_ obtained for APE_O_-g-PiPrOx in NP (Figure 5), which, at close *M*_w_ of all copolymers, could be caused by swelling of PiPrOx side chains in this solvent. However, the increase in *R*_h_ by more than 2 times in DMF rather attributes to intermolecular aggregation, what is indicated by a negative *k*’ value. This issue will be discussed below.

Based on the experimentally measured values of *M*_w_ for APE_O_ (*M*_APE_) and the copolymers, as well as the mass of the side chain *M*_s_, the architecture parameters of the APE_O_-g-PiPrOx graft copolymer were calculated, which are given in Table 2. The side chain length in the extended conformation without deformation of bond angles was calculated using the formula *L*_s_ = λ × *N*_s_, since the length of the projection of the alkylene unit onto the direction of the PiPrOx chain is λ = 0.252 nm. Accordingly, molar masses *M*_s_ of side chains are equal to *M*_s_ = *N*_s_ × *M*_0s_, where the mass of the PiPrOx monomer unit, *M*_0s_ = 113 g/mol. Then the degree of functionalization of the macroinitiator (or grafting density of side chains) is *z* = *m/(n  +  m*) = *m*/*N*_0_, where *m* is the number of the backbone monomer units containing side chains (i.e., the number of side chains) and *n* is the number of the monomer units of the macroinitiator which do not contain side chains [59,60]; *m* was calculated using the formula *m* = (*M*_w_ − *M*_APE_)/*M*_s_.

It can be seen from Table 2 that in all cases the value of *z* is small, i.e., APE_O_-g-PiPrOx macromolecules are brushes with rather loosely attached side chains. Less than half of the monomer units in the main chain carry the side chains. The value of *z* decreases upon an increase in *L*_s_, and for sample 1 this is about two times greater than for samples 2 and 3. At the same time, the side chains are 2.2–2.7 times longer than the distance between the grafting points, calculated as ∆*L* = *L*_0_/*z* (Table 2). The parameter *L*_s_/∆*L* makes it possible to compare the distance between the grafting points of the side chains and their length, which is maximum for sample 1.

The degree of shielding of the molecular brush backbone by side chains and its accessibility for contacts can be quantitatively characterized by the parameter δ that takes into account the size of the side chain folded into a molecular coil [24]. This parameter is conveniently calculated in terms of the number of bonds in the side chains and in the fragments of the main chain between their grafting points, *δ* = [(*N*_A_ × *N*_s_)]^0.5^/(*N*_0_ × z), where the number of bonds in the Kuhn segment of the side chain *N*_A_ = 3 for PiPrOx [74], and *N*_0_ × *z* characterizes the number of bonds in the backbone of the brush between adjacent side chains. Characteristically, the degree of shielding calculated for the studied brushes is always low (Table 2). However, the parameter *δ* obtained for brush 1 significantly exceeds this parameter for brushes 2 and 3 with longer side chains, but with a lower side chain grafting density.

### 3.4. Composition of Scattering Species in Solution and Their Hydrodynamic Size at Room Temperature

**Figure 6:**
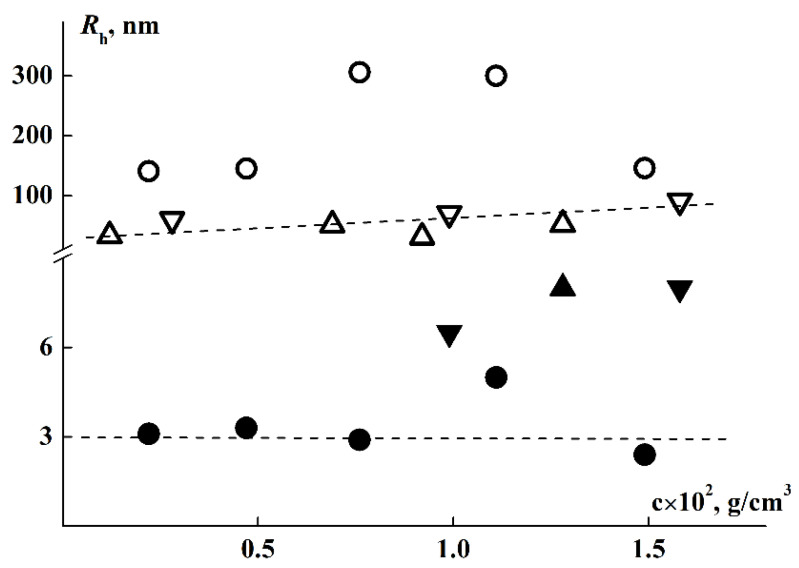
Concentration dependence of *R*_f_ (solid) and *R*_s_ (open) for copolymers 1 (circle), 2 (triangles), and 3 (inverse triangles) in water at T = 21 °C.

At room temperatures (*T* < 27 °C), two modes associated with scattering species with hydrodynamic radii *R*_f_ and *R*_s_, were registered in DLS experiments in water solutions. For all samples, species of large hydrodynamic size, which are aggregates, were observed. Their averaged hydrodynamic radii *R*_s_ were 300 nm for brush 1, 40 nm for brush 2 and 170 nm for brush 3 (Table 1). It can be noted that *R*_s_ tends to increase with concentration despite a general significant scatter of points (Figure 6). The proportion of light scattered by aggregates, *S*_s_, in the total light scattering always exceeds 98.5%. However, it should be understood that a high contribution from large species to scattering intensity does not mean their high concentration in solution. Indeed, for a particle of type i, the relation I_i_ ~ *c*_i_*M*_i_ is true, where *M*_i_ and *c*_i_ are the molar mass of the specie and its content in the solution. In this case, M_i_ ~ R_i_^x^, where the parameter x depends on the particle shape. For example, x = 1, 2 and 3 for the long rod, the macromolecular coil, and for the sphere, respectively. Then the partial concentration for spheres is c_i_ ~ *I*_i_/*R*_i_^3^. It can be estimated that for spherical particles, whose sizes differ by 1–2 orders, even at a high contribution of the scattering intensity, the concentration *c*_i_ can be very small. However, since the shape and density of aggregates are a priori unknown, we cannot obtain their content in solution. Though measurements at a large scattering angle could be advantageous when using DLS to determine the relative concentration of nanoparticles in the samples with bimodal size distributions [76], the question of the physical and topological properties of supramolecular structures does not allow us to carry out a quantitative analysis of their content in solution. Nevertheless, we can trace the change in the contribution of various species to light scattering with a change in the external parameters of the medium, such as concentration, temperature, etc. 

From Table 1 it follows that the size of the fast mode *R*_f_, obtained for aqueous solutions, exceed the radii of macromolecules *R*_h_, determined in organic solvents. On the one hand, this may indicate increased size of macromolecules caused by swelling of PiPrOx chains in water, which apparently occurs in the case of brush 1. On the other hand, with a loose attachment of side chains, it is more likely that even far from the phase separation temperatures, the macromolecules form small supramolecular structures. As was shown above, the degree of shielding of the main chain by side chains is low for the studied brushes, especially in copolymers 2 and 3, which gives preconditions for hydrophobic interactions of their main chains and hence their intermolecular aggregation. 

### 3.5. Thermoresponsiveness of APE_O_-g-PiPrOx in Water While Heated

**Figure 7:**
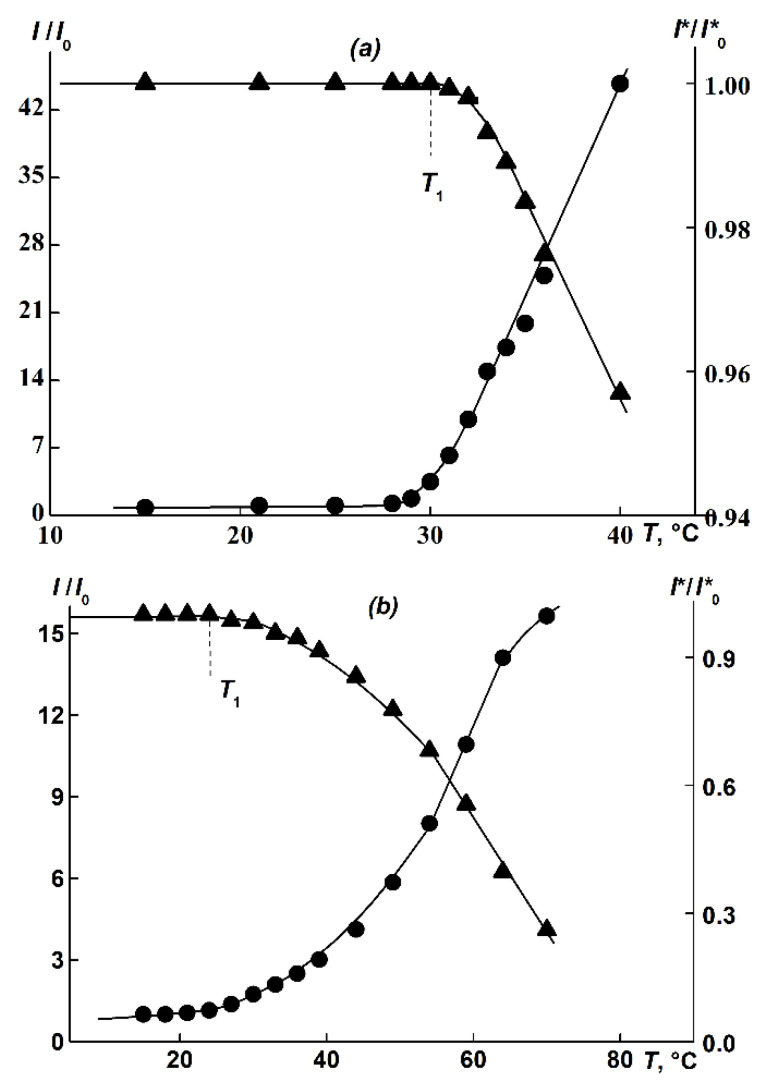
*I/I*_0_(*T*) and I*/I*_0_(*T*) for sample 1 (**a**) at *c* = 0.0022 g/cm^3^ and sample 3 (**b**) at *c* = 0.016 g/cm^3^.

Upon heating APE_O_-g-PiPrOx aqueous solutions, a significant increase in *I* and, respectively, a decrease in optical transmission I* are observed, indicating a phase separation (Figure 7). For copolymer 1, the increase in *I* was quite sharp. However, a smooth appearance in *I/I_0_(T)* and I*/I*_0_(T) dependencies, where *I*_0_ and I*_0_ are initial scattering intensity and optical transmission at *T* = 15 °C, respectively, for brush solutions 2 and 3 were obtained. Similarly, a smooth augmentation in *I* was noted in the study of the PEtOx graft copolymer synthesized on a flexible-chain aromatic macroinitiator of a similar structure APE_6_-g-PEtOx containing an alkylene spacer [60,61]. The temperature *T*_1_ was determined from the beginning of the drop in the optical transmission I* (Figure 7).

**Figure 8:**
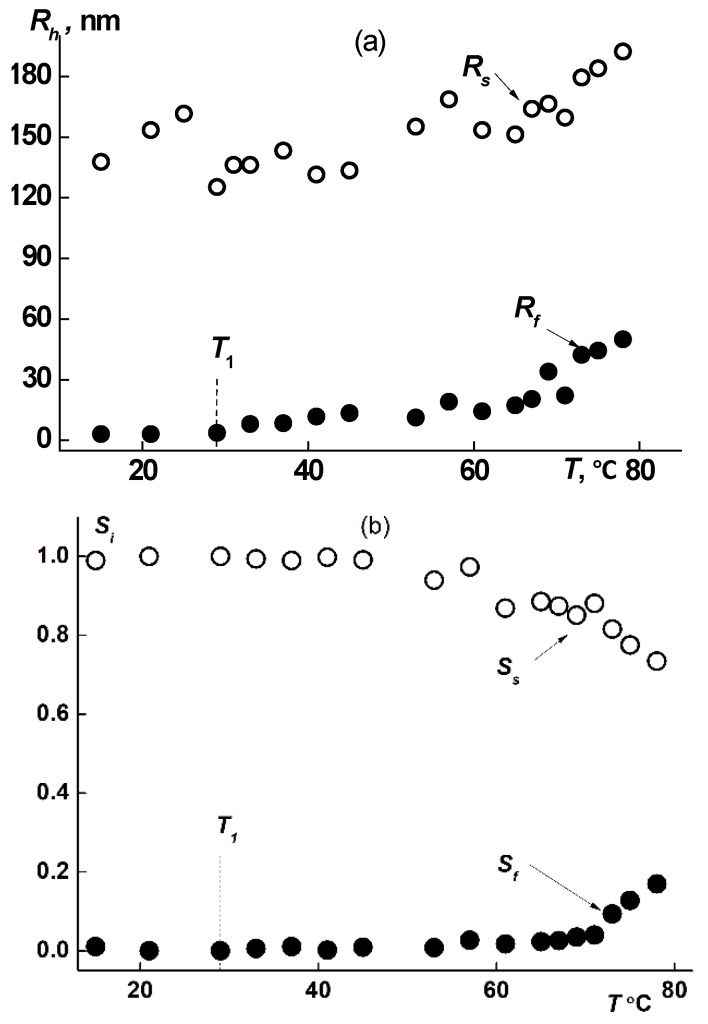
Progress of hydrodynamic size (**a**) and *S*_i_ (**b**) (i is f or s) upon heating for fast and slow modes in sample 1 aqueous solution at c = 0.0047 g/cm^3^.

**Figure 9:**
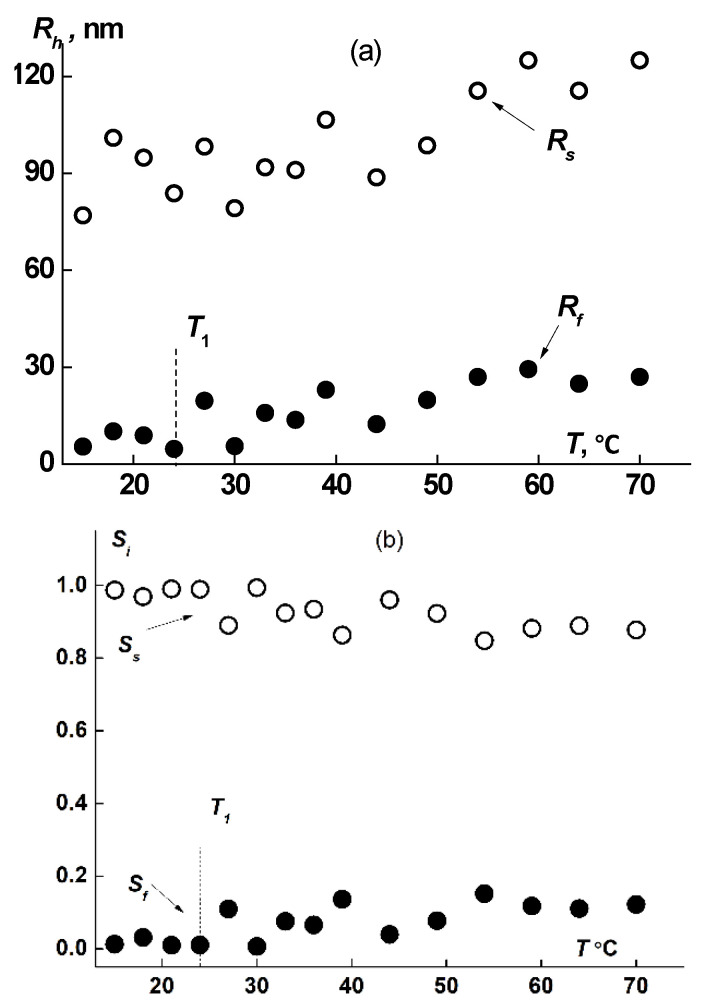
Progress of hydrodynamic size (**a**) and *S*i (**b**) (i is f or s) upon heating for fast and slow modes in sample 3 aqueous solution at c = 0.016 g/cm^3^.

The hydrodynamic dimensions of dissolved species of both types, *R*_f_ and *R*_s_, in solutions of all samples increased upon heating (Figure 8a and Figure 9a), augmentation beginning in the vicinity of *T*_1_. The increase in size takes place due to attaching of macromolecules to aggregates and to coupling of existing supramolecular structures. Thus, the fast mode in the sample 1 aqueous solutions transforms from macromolecules to small aggregates. The aggregate formation upon heating is conditioned by the decrease of PiPrOx solubility due to dehydration of 2-isopropyloxazoline units, which begins already at *T* ≈ 20 °C [63]. Note that the relative rate of change in the size of small aggregates *R*_f_ is noticeably higher than the rate of increase in *R*_s_. Thus, *R*_f_ increases almost 20 times when heated to T ≈ 80 °C, while *R*_s_ changes by approximately 30%. Accordingly, the contribution of large aggregates to light scattering *S*_s_ at temperatures above *T*_1_ decreases, while *S*_f_ increases (Figure 8b and Figure 9b).

### 3.6. Concentration Dependence of the Phase Separation Temperatures

**Figure 10:**
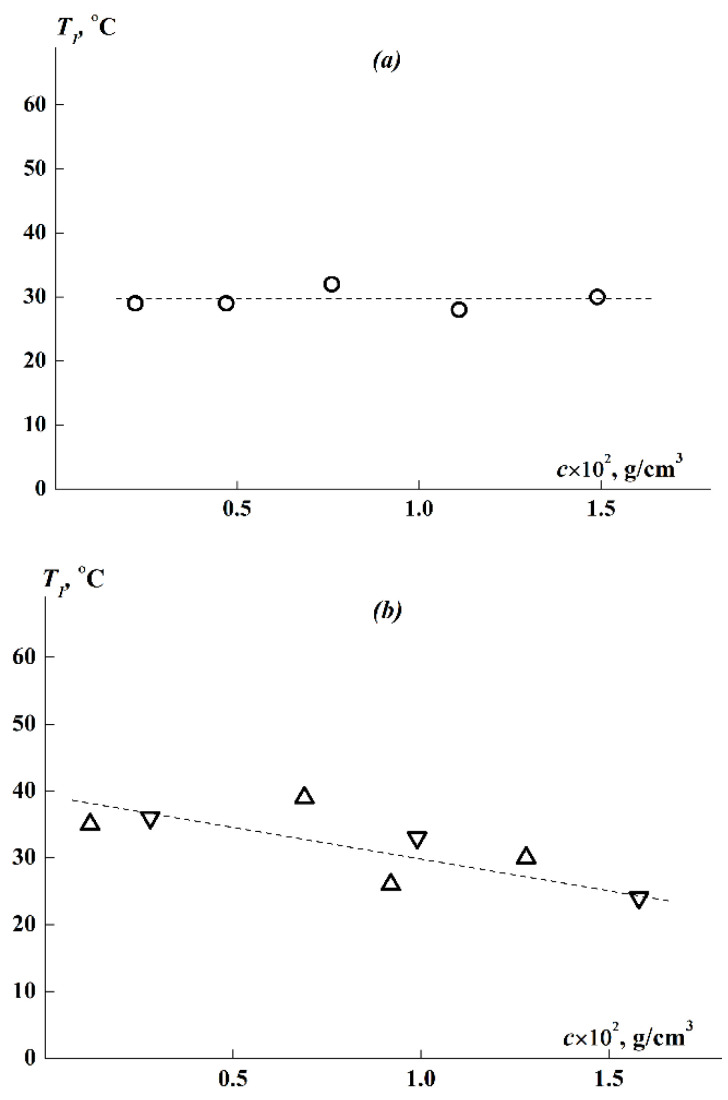
Concentrational dependence of phase separation temperatures for (**a**) sample 1 and (**b**) samples 2 (triangles) and 3 (inverted triangles) in APE_O_-g-PiPrOx aqueous solutions.

The *T*_1_ values obtained for APE_O_-g-PiPrOx at various concentrations are generally in the same region (Figure 10a,b). The *T*_1_ for brush 1 with shorter side chains and higher *z* is practically independent of concentration, and the concentration-averaged temperature of the phase transition is <*T*_1_> = (30 ± 1) °C. As for brushes 2 and 3, despite the rather large scatter of experimental points, they fit into the general dependence *T*_1_(c), demonstrating a tendency for *T*_1_ to decrease from 35 to 24 °C with an increase in *c* within a more than 10-fold concentration range. This trend is consistent with the previously obtained dependence for PiPrOx molecular brushes with flexible and rigid main chains [62,65]. In this case, in general, the region of the onset of phase separation *T*_1_ for the studied APE_O_-g-PiPrOx lies above the entire corresponding temperature range 20–25 °C for the phase separation of graft copolymer with a flexible backbone APE_8_-g-PiPrOx [62].

The above results show similar behavior of copolymers with longer side chains but lower *z* in solutions at room temperature and upon heating: the *I/I*_0_(c) profile, the composition and size of the scattering particles, and the phase separation temperatures as functions of concentration are similar for samples 2 and 3. This can undoubtedly be due to their similar molecular properties, which can be described using architectural parameters, such as *z*, *L*_s_, δ and so on.

## 4. Discussion and Conclusions

**Figure 11:**
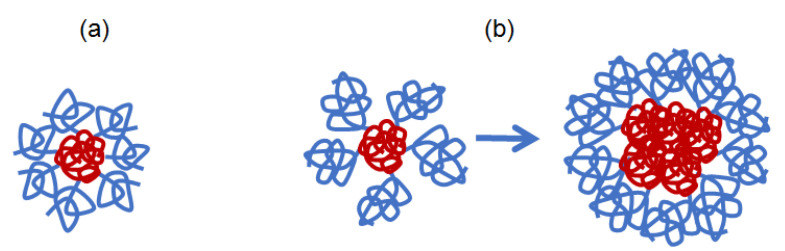
Model conformations for APE_O_-g-PiPrOx samples 1 (**a**) and 2 and 3 (**b**) in water.

The studied APE_O_-g-PiPrOx copolymers with blocks that differ in affinity to the solvents, at low *z* exist in the regime of strong intra- and intermolecular interactions. For side chains of different length and similar backbone, their behavior can be controlled by the parameter δ, which is a parameter to compare the distance between the grafting points on the macromolecule backbone (and hence, *z*) and the model dimensions of the side chains, taking into account the size and rigidity of their structural units. The studied copolymers exhibiting similar molecular and thermoresponsive properties, namely samples 2 and 3, are characterized by a close value of δ, which differs by about 1.5 times from the δ value for sample 1 (Table 2). Due to the ability of the semirigid APE_O_ to change the conformation, the main chain is always folded to avoid the solvent and is surrounded by swollen side chains. Schematically, the considered macromolecules with the same length of the main chain but different side chain length and different low grafting degree can be represented as star-shaped structures, with a core of collapsed APE_O_ chain, and loosely grafted PiPrOx arms which are in a coiled conformation. In the case of sample 1, where the degree of shielding is δ = 0.21, the macromolecules are soluble, similarly to that for APE_8_-g-PiPrOx with flexible backbone [62] (Figure 11a). At δ ≤ 0.14 (samples 2 and 3), molecular coils of a soluble PiPrOx do not shield the core sufficiently, which creates the preconditions for the aggregation of copolymers due to hydrophobic interactions of the main chains (Figure 11b). For comparison, for the brush APE_8_-g-PiPrOx with long flexible –(CH_2_)_8_– spacer in the main chain and rather short PiPrOx arms (*N*_s_ = 37), the calculated value of δ = 0.18 is close to that of sample 1 (Table 3).

Since we determined the transition temperatures *T*_1_ over a wide, more than 10-fold concentration range from 0.0012 to 0.016 g/cm^3^ (see Figure 10), we attempted to estimate the LCST for the APE_O_-g-PiPrOx samples. For sample 1, whose solutions did not demonstrate a noticeable dependence *T*_1_(*c*), we can apparently assume LCST ≈ <*T*_1_> ≈ 30 °C. For sufficiently hydrophobic samples 2 and 3, the value of *T*_1_ decreases monotonically with increasing concentration. Judging by our observations of the dissolution of the samples, it is unlikely that, with a further increase in c above the measurement interval, the solubility of the polymer will improve, and hence *T*_1_ will increase. Therefore, LCST < 24 °C for samples 2 and 3 can be estimated. It should be understood that the LCST estimation performed for APE_O_-g-PiPrOx is rather rough, but it is helpful for comparing the thermosensitivity of polyoxazoline brushes with aromatic polyester backbones of different rigidities (Table 3).

Table 3 represents parameters for PiPrOx grafted copolymers with APE main chain of different rigidity. Due to the high proportion of the hydrophobic component ω = *M*/*M*_w_ in the composition of APE_O_-g-PiPrOx, where *M* = *M*_w_ − *M*_APE_ (Taблe 3), its hydrophilic–hydrophobic balance in water solutions is shifted to the temperature below the LCST detected for linear PiPrOx. As discussed in the Introduction, the cloud points for PiPrOx were observed at 36 < T < 63 °C depending on concentration, molar mass and architecture [40,41,64]. For block copolymers that are identical in chemical structure, but with a difference in architectural parameters, at close values of δ, a similar behavior of the APE_O_ copolymers upon heating is observed; at higher δ, the LCST of the polymer is higher. However, upon transition to a rigid aromatic polyester main chain, the thermoresponsiveness range is significantly lower, than it is for APE_O_-g-PiPrOx at close structural parameters. Thus, the ability of the main chain of the grafted block copolymer to undergo intramolecular transformations plays a significant role in the formation of its properties in solutions at room temperature and upon heating.

## Figures and Tables

**Table 1:** Molecular characteristics for APE_O_ and APE_O_-g-PiPrOx.

Sample	Initiator/ Monomer	Solvent	[η], cm^3^/g	*k*′	*dn/dc*, cm^3^/g	*M*_w_, g/mol	*A*_2_·10^4^, cm^3^ mol/g^2^	*R*_h,_ nm	in Water at 21 °C	
									*R*_f_, nm	*R*_s_, nm
APE_O_		CHCl_3_	53	0.33	0.176	22,000	3	1.5	-	-
1	1/40	NP	3.0	1.46	0.079	71,400	13	1.6	2.5	145
2	1/100	NP	2.4	12	0.072	62,800	2.7	2.4	8	42
3	1/120	NP			0.072	68,000	~0	2.7	12	78
		DMF	9.6	−0.8				6.0		

**Table 2:** Structural parameters for APE_O_-g-PiPrOx.

Copolymer	*M*_w_, g/mol	*M_s_*	*z*	*L*_s_, nm	Δ*L,* nm	*L_s_*/Δ*L*	δ
***1***	71,400	3400	0.38	11.3	4.2	2.7	0.21
***2***	62,800	5650	0.19	18.8	8.4	2.2	0.14
***3***	67,000	6800	0.17	22.6	9.1	2.5	0.13

**Table 3:** Comparison of PiPrOx bottle brushes with different rigidity of the APE main chains.

Copolymer	*N_0_*	*A*, nm	*N*_s_	*z*	δ	ω	LCST, °C
1	38	2–4	30	0.38	0.21	0.31	30
2	38	2–4	50	0.19	0.14	0.35	<24
3	38	2–4	60	0.17	0.13	0.33	<24
APE_8_-g-PiPrOx [62]	27	2	38	0.49	0.18	0.26	20
APE_r.ch._-g-PiPrOx [65]	47	28	30	0.27	0.2	0.5	<20

## Data Availability

Not applicable.

## Supplementary Materials

The following supporting information can be downloaded at: https://www.mdpi.com/article/10.3390/polym14245354/s1, Figure S1: Dependences cH/Iex(c) for APEO-g-PiPrOx solutions of samples 1 (a), 2 (b) and 3 (c) in nitropropane.

## Abbreviations

APE_O_	aromatic polyester with—O—spacer
APE_6_	aromatic polyester with—(CH_2_)_6_—spacer
APE_8_	aromatic polyester with—(CH_2_)_8_—spacer
APE_r.ch._	rigid chain aromatic polyester
*M*_w_	weight-average molar mass
*M*_0_	the mass of the macroinitiator monomer unit
*M*_APE_	the macroinitiator molar mass
*N*_0_	the degree of polymerization of the macroinitiator
*L*_0_	the length of the monomer unit of the macroinitiator
*L*	the length of the macroinitiator macromolecule
*A*	the Kuhn segment length
*M*_s_	the mass of the side chain
*N*_s_	the degree of polymerization side chains
*M*_0s_	the mass of the side chain monomer unit
λ	the length of the projection of the alkylene bond onto the direction of the PiPrOx chain
*n*	the number of side chains
*z*	the density of side chains grafting
Δ*L*	the distance between the grafting points along the main chain
δ	the degree of shielding of the backbone by side chains
