# Supplementary material for: Loose Semirigid Aromatic Polyester Bottle Brushes at Poly(2-isopropyl-2-oxazoline) Side Chains of Various Lengths: Behavior in Solutions and Thermoresponsiveness
Source: Polymers (Basel). 2022 Dec 7;14(24):5354. doi: 10.3390/polym14245354 (PMC9781464; doi:10.3390/polym14245354)
Supplement: Supplementary file 1 [file polymers-14-05354-s001.zip › polymers-2055024-supplementary.pdf]

Supplementary information

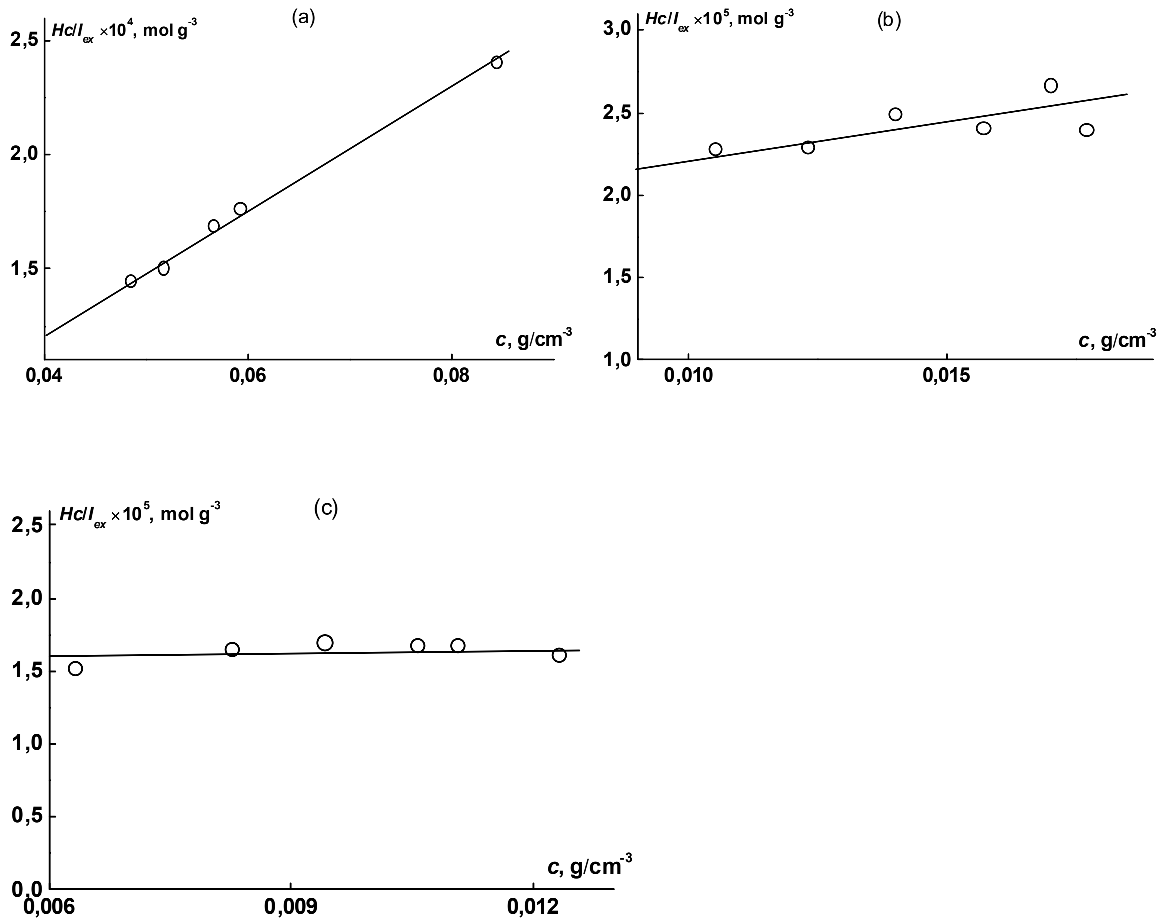

Figure S1. Dependences cH/I_{ex}(c) for APE_O-g-PiPrOx solutions of samples 1 (a), 2 (b) and 3 (c) in nitropropane
